# Ageing Population and Balance Under Stressful Conditions—A Cross-Sectional Observational Study

**DOI:** 10.3390/healthcare14020237

**Published:** 2026-01-18

**Authors:** Isabel Rodríguez-Costa, Belén Díaz-Pulido, Yolanda Pérez-Martín, Susana Nunez-Nagy, Miguel Ángel Valero-Gil, Alejandra Cano-Hernamperez, Sara Trapero-Asenjo

**Affiliations:** 1 Department of Nursing and Physiotherapy, Faculty of Medicine and Health Sciences, University of Alcalá, 28805 Alcalá de Henares, Spain; isabel.rodriguezc@uah.es (I.R.-C.); belen.diazp@uah.es (B.D.-P.); susana.nunez@uah.es (S.N.-N.); sara.trapero@uah.es (S.T.-A.); 2 Humanization in the Intervention of Physiotherapy for the Integral Attention to the People Group (HIPATIA), University of Alcalá, 28805 Alcalá de Henares, Spain; 3 Health Technology Integration Research Group (GITES), Castilla-la Mancha Health Research Institute, 45004 Toledo, Spain; 4 Faculty of Medicine and Health Sciences, University of Alcalá, 28801 Alcalá de Henares, Spain; miguel.valero@edu.uah.es (M.Á.V.-G.); alejandra.canoh@edu.uah.es (A.C.-H.)

**Keywords:** aged, balance, risk factors, stress, psychological, emotions, psychomotor performance, autonomic nervous system

## Abstract

**Background/Objectives**: Falls are a major global issue for older adults, and emotional stress may increase the risk due to its effects on postural control and balance. However, the immediate effects of a stressful stimulus on objective measures of balance and fall risk are unknown. The study aims to explore differences in older adults’ performance on the Timed Up and Go (TUG) test before and after such exposure. **Methods**: In this cross-sectional study, 31 older adults (71.6 ± 4.98 years) were exposed to an emotionally stressful stimulus using high-arousal images from the International Affective Picture System. Participants performed the TUG before (t1) and after (t2) exposure as the primary outcome measure. To assess the physiological and psychological impact of the stressful stimulus, heart rate variability (HRV) was recorded before and during image viewing. A visual analogue scale (VAS) of unease was completed both before and after the stimulus. **Results**: During the stressful stimulus, the HRV high-frequency (HF) band decreased significantly (*p* = 0.001), while the low-frequency (LF) band (*p* = 0.002) and the LF/HF ratio (*p* = 0.004) showed a significant increase. Similarly, after stressful stimulus, VAS scores demonstrated a statistically significant increase (*p* < 0.001). The time to complete the TUG showed a statistically significant increase at t2 (*p* < 0.001). **Conclusions**: The stressful stimulus triggered both physiological and subjective stress responses. Subsequently, TUG test performance declined (increased duration), suggesting that emotionally stressful stimuli could deteriorate functional balance performance in older adults, potentially increasing fall risk.

## 1. Introduction

In many nations, the number of people over 60 has been rising over the past few decades. Furthermore, it is estimated that there will be two billion senior individuals worldwide by 2050 [1]. The body undergoes several changes in organs and tissues during senescence, including a decline in activity, which may indicate geriatric syndromes, a higher risk of falls, and related frailty [2]. A fall is defined by the World Health Organization (WHO) as “an involuntary event which results in loss-of-balance bringing the body to the ground or other surface.”In this regard, people over 60 havethe highest fatality rates related to falls [3].

Falls are the second greatest cause of death globally and can be one of the negative consequences of frailty in the elderly [4,5]. In addition to hospitalization, immobility, and the ensuing decline, falls in the elderly typically result in dread of loss of balance and falling again, which can further cause disability [6,7]. The natural aging process is frequently associated with a decline in balance and postural control, which can lead to major health issues [8,9]. Sensorimotor processes are impacted by falls in the elderly [10,11]. A study developed in Spain with adults over the age of 65 found that 51.7% of participants had alterations in one of the sensory afferent systems (visual, vestibular, and somatosensory), 25.3% in two of the afferent systems, and 11.1% in all three sensory systems, highlighting the deterioration of these systems with age [10,12,13].

Neuropsychological factors, depression, anxiety, and stress contribute to further degradation of sensory-motor functioning [11]. Postural threat and passive viewing of aversive or threatening images affect postural control [14], thereby reducing postural balance. In this regard, Johnson et al. [15] suggest that changes in postural control in relation to postural threat are closely connectedto the emotional response to the threat itself. Consistent with this, autonomic nervous system function was worse in older fallers [16].

The study by Möller et al. [17] retrospectively examined whether patients over the age of 65 with hip or pelvic fractures had experienced emotional stress before the fracture. The results showed an increased risk of falling up to one hour after an episode of emotional stress [17]. In addition, Akinlosotu et al. [18] demonstrated that cognitive load and mental stress reduce the effectiveness of balance maintenance responses in older adults. However, the acute and immediate effects of exposure to a stressful emotional stimulus on objective metrics of balance and fall risk in the elderly population have not yet been clearly elucidated.

One of the tests widely used in older adults to measure balance and to predict falls is the Timed Up and Go (TUG) test [7,19]. The TUG has been demonstrated to have validity [20,21] and correlates with the Berg Balance Scale [22] and gait speed [23]; consequently, it is frequently employed in the investigation of older populations [4]. Additionally, theTUG can distinguish between different fall risks [24,25]. The TUG measures the time required (in seconds) for a person to rise from a chair with armrests, walk three meters using their usual assistive devices, turn, return to the chair, and sit down [21]. Although the most popular form of the TUG requires participants to walk at a comfortable pace [21], other modified variants have been created that involve walking as quickly as possible [26] and incorporate cognitive and motor tasks [25,27]. The relationship between physical performance and autonomic function in older fallers can be observed using the TUG [16].

Considering that exposure to a stressful emotional stimulus through image viewing affects postural control [14] and that mental stress reduces the effectiveness of the responses necessary to maintain balance [18], we hypothesize that exposure to a stressful stimulus will increase the time required to complete the fast-paced TUG, which reflects dynamic functional mobility and fall risk in older functional adults. This study aims to explore differences in TUG performance in older adults before and after exposure to a stressful emotional stimulus, examining whether acute emotional stress transiently impairs postural control and dynamic mobility as evidenced by longer TUG completion times.

## 2. Materials and Methods

### 2.1. Study Design

This is a cross-sectional observational study conducted in accordance with the principles of the Declaration of Helsinki and approved by the Ethics Committee for Research and Animal Experimentation of the University of Alcalá (CEIP/2023/5/110). This study is part of the research project “Use of Devices for Detecting Balance in Older Adults under Stress Influence—DEPIE”, which has been registered on ClinicalTrials.gov under the code NCT06682754.

### 2.2. Participants

The participants were older adults aged 65 or older who voluntarily decided to participate in the study after reading the information sheet and signing the informed consent form. The exclusion criteria included: difficulty understanding the study information; illness, injury, or previous trauma that contraindicates muscle exertion, balance exercises, and/or walking; and physical or mental illness that contraindicates exposure to emotionally stressful stimuli, such as severe depression or psychosis.

The sample size was calculated using G*Power (version 3.1.9.7), setting a significance level (α) of 0.05, with a statistical power (1 − β) of 0.85 and assuming an effect size (d_z)_ de 0.6, based on previous studies [18]. Thus, the required sample size was 31 participants. The reference study by Akinlosotu et al. [18] determined that an effect size of 0.6 was sufficient to detect significant changes in postural reactions under stressful conditions in older adults.

All participants were recruited from February to July 2025 using non-probability sampling through contacts with the University of Alcalá’s University for Seniors, the City Council, and associations in Alcalá de Henares.

### 2.3. Materials and Variables

During the experiment, participants executed the TUG test as the primary outcome measure on two occasions: prior to and following the stressful emotional stimulus. The TUG measures the duration required for a person to rise from a chair, walk three meters, turn, and return to the chair to sit [21]. Three trials are performed, and the average time is then calculated. Longer times indicate a higher risk of falls [28].

The stressful emotional stimulus, on the other hand, was the viewing of high-arousal images from the International Affective Picture System (IAPS). The IAPS [29] is one of the most widely used sets of pictures for inducing emotions in experimental research. Exposure to these images has been shown to produce changes across the three emotional response systems: subjective-verbal, behavioral, and physiological [30]. For this study, 30 high-arousal images from IAPS were used (3530, 2683, 9181, 6571, 2352.2, 9635.1, 3230, 6020, 3550, 6370, 9433, 9810, 9435, 6570, 9050, 1300, 9400, 9622, 6831, 6560, 9250, 9140, 9265, 6244, 1525, 2800, 6555, 9571, 9430, 3301). These produce an average arousal score of 7.826 in older adults according to the normative values of previous studies [31]. Each image was displayed consecutively for 6 s over a total duration of 3 min.

To confirm that stress had occurred during the viewing of the IAPS images, Heart Rate Variability (HRV) and subjective feelings of unease were collected as secondary outcome measures. On the one hand, the physiological data for HRV calculation were recorded using the Hexoskin Pro vest (Hexoskin Inc., Montreal, QC, Canada). Subsequently, these data were analyzed in the frequency domain using Kubios HRV Premium software version 3.5.0 [32]. The low-frequency (LF) (0.04–0.15 Hz) and high-frequency (HF) (0.15–0.4 Hz) bands were calculated [33]. The HF band reflects parasympathetic modulation, and the LF band is modulated by both the sympathetic and parasympathetic nervous systems [34,35,36]. The LF/HF ratio was also calculated as an index of sympathetic-vagal balance. The HRV was evaluated in 3 min periods, which provided sufficient duration to capture both LF and HF components in accordance with minimum standard requirements [33,35,36]. Previously, a 10 min stabilization period was performed to allow participants to reach a baseline physiological state before recording. These physiological variables were measured before and during the viewing of the IAPS images. Participants were also asked to complete a Visual Analogue Scale (VAS) before and after viewing the IAPS images. The VAS consists of a line ranging from 0 to 10. In this case, 0 represented “completely relaxed” and 10 represented “the greatest feeling of unease, restlessness, or anxiety you have ever felt.” To complete the scale, participants were asked to mark the point on the line between the two extremes that best reflected their emotions at that moment.

### 2.4. Procedure

A single session was performed. All sessions were scheduled during the morning. Data collection was conducted in a designated room at the University of Alcalá and was developed by one of the researchers. The room temperature remained constant throughout all sessions. Initially, the participants’ sociodemographic data were collected, and they were fitted with the Hexoskin Pro vest. Subsequently, all participants executed the TUG for the first time (t1) with instructions to perform the test as quickly as possible while feeling safe [27]. Then, they were seated in a chair and remained for 10 min. Following this, physiological baseline measurements were collected for 3 min in that position, and the participants completed the VAS (pre-stress). Next, the participants viewed 3 min of high-arousal IAPS images while seated, and physiological measurements were taken. Subsequently, they completed the VAS (stress). Finally, the participants executed the TUG for the second time (t2) with instructions to do it as quickly as possible while feeling safe. After that, the Hexoskin Pro vest was removed.

### 2.5. Statistical Analysis

A different researcher from the one who collected the data performed the statistical analyses using the free software RStudio version 2024.09.0.

First, descriptive statistics were calculated for the qualitative variables (sex, residential status, education level, and physical activity level) and quantitative variables (age, time of day, LF, HF, LF/HF, VAS, and TUG). Education level was categorized into five groups: no education, primary education, secondary education, technical education, and university education. Physical activity levels were classified according to World Health Organization guidelines [37]. Participants in the low category did not meet the minimum recommendation of 150 min of moderate-intensity aerobic activity per week; the moderate group met these baseline requirements (150–300 min); and the high category exceeded 300 min of weekly exercise, representing a high level of functional engagement. Qualitative variables were summarized using frequencies and percentages. Quantitative variables were summarized using the mean and standard deviation (M ± SD) or the median and interquartile range (Md [Q1; Q3]), depending on their distribution. The normality of the distribution of quantitative variables was assessed using the Shapiro-Wilk test. Variables that did not meet the normality assumption were explored for correction using Box-Cox logarithmic transformations.

Changes in stress induction (comparing ‘pre-stress’ versus ‘stress’ moments) using the LF, HF, LF/HF, and VAS variables, and the changes in the TUG (comparing ‘t1’ versus ‘t2’moments) were analyzed. The student’s *t*-test for dependent (paired) samples was applied when the variables met the assumption of normality. When the absence of normality could be corrected using logarithmic transformations, these transformations were implemented to enable parametric analyses. The Wilcoxon signed-rank test for dependent samples was employed when the variables did not follow a normal distribution and the logarithmic transformation was not sufficient to correct the asymmetry. A significance level (α) of 0.05 was established for all analyses, and 95% confidence intervals (CI) were calculated for all mean and median differences. In all cases, the effect size was calculated to quantify the magnitude of the difference: for the student’s *t*-test, Cohen’s d was calculated, and its values were interpreted as small (0.2), medium (0.5), or large (≥0.8). For the Wilcoxon test, the r was calculated, where values were interpreted as small (0.1), medium (0.3), or large (≥0.5).

## 3. Results

The sample consisted of 31 participants (71.6 ± 4.98 years), including 7 (22.58%) men and 24 (77.42%) women. All participants were non-institutionalized and lived in the community. In terms of education level, the participants’ distribution was as follows: 14 (45.17%) had primary education, 6 (19.35%) had no education, 6 (19.35%) had technical education, 3 (9.68%) had university education, and 2 (6.45%) had secondary education. Regarding physical activity levels, 15 (48.38%) participants were categorized as high, whereas 8 (25.81%) were classified as moderate and 8 (25.81%) as low. Finally, experimental sessions were conducted at 11:12 [9:47; 11:30] hours.

### 3.1. Stress Induction

To assess the impact of the stressful stimulus on autonomic regulation and subjective experience, heart rate variability and perceived unease were analyzed. The results showed a significant decrease in the HF band, as well as a statistically significant increase in the LF band, the LF/HF ratio, and the VAS of unease (Table 1), indicating a shift towards a decrease in the predominance of the parasympathetic dominance during stress induction. These changes suggest that the emotionally stressful stimulus effectively triggered a physiological response to stress and an increased perception of unease in older adults.

### 3.2. Timed Upand Go Test

**Figure 1:**
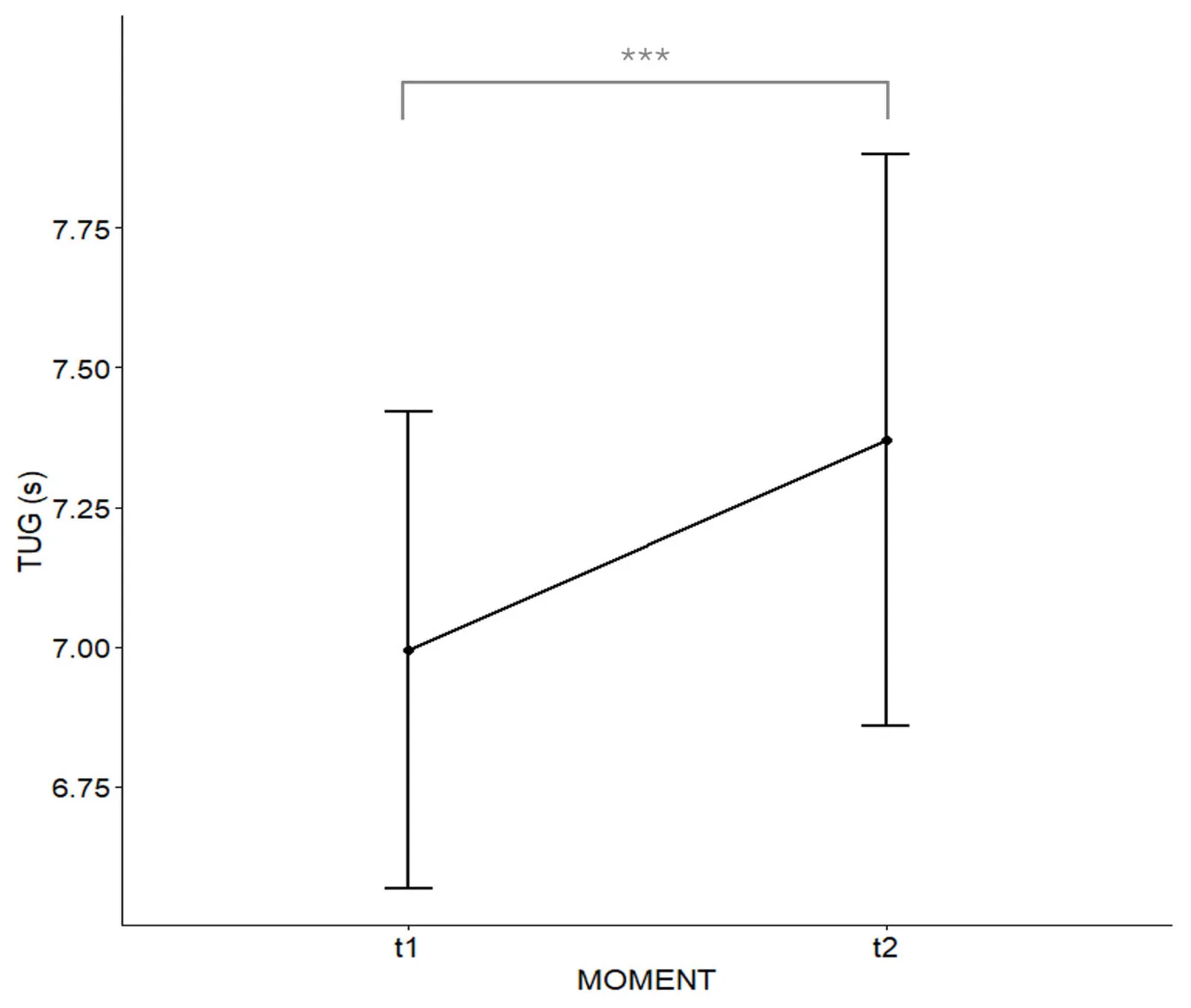
Evolution of Timed Up and Go (TUG) test times at moments t1 (before the stressful emotional stimulus) and t2 (after the stimulus), showing the increase in TUG time at t2. The dots represent the mean values, and the error bars represent the 95% confidence interval. The significant difference of *p* < 0.001 obtained between moments is represented by three asterisks (***).

To evaluate the impact of the stressful stimulus on dynamic functional mobility, TUG performance was compared between baseline (t1) and post-stress (t2) conditions. The TUG test showed a statistically significant change between the two measurement moments (t1 vs. t2). The mean execution time for the TUG increased significantly from t1 (7.00 ± 1.16 s) to t2 (7.37 ± 1.40 s), t(30)= −4.23, 95% CI [−0.54, −0.20], *p* < 0.001, d=−0.76 (Figure 1). This 0.37 s deterioration in TUG performance indicates that acute emotional stress may compromise dynamic balance and functional mobility in older adults.

## 4. Discussion

The study aimed to explore differences in TUG performance in older adults before and after exposure to a stressful emotional stimulus. The results of the secondary outcome variables indicated successful stress induction, with a significant decrease in parasympathetic dominance, as well as a clear increase in the subjective feeling of unease. The Timed Up and Go (TUG) test analyses showed significantly longer times at time t2. Specifically, the average TUG time increased from 7.00 s to 7.37 s, representing a slowdown of 0.37 s, and indicating that the time required to complete the test increased after the stressful emotional stimulus.

The initial hypothesis of the research was that exposure to a stressful stimulus would increase the time to complete the TUG test. As other research shows [38], maintaining postural control and balance requires a regulation of the motor, sensory, and cognitive processes that can be affected by emotional stimuli. These disturbances can influence postural sway and promote a decrease in body movements in response to emotionally charged, frightening input [14,39,40]. This acute mental stress or emotional threat can induce a “freezing” strategy or postural rigidity, which is maladaptive for dynamic, quick-reaction tasks such as the TUG [11,14], explaining the increase in the time needed to fulfill the test. This stiffening reaction promotes smaller balance corrective steps and is less capable of enhancing protective postural reactions following repeated disruptions [40].

Literature generally agrees that at least two distinct emotion-specific paradigms, postural threat, and passive watching of unpleasant or frightening images can cause fear-related postural reactions [14]. This kind of response is consistent with research on balance threats provoked with threatening or aversive images, which observes a typical “freezing” or “stiffening” mechanism in reaction to heightened stress, anxiety, and threat [41,42]. Furthermore, this “freezing” mechanism is associated with changes in breathing [41], and previous studies have reported changes in postural control related to breathing and alterations in spontaneous breathing [43,44].

These strategies can promote a vicious circle [45]. The study by Carpenter et al. [46] demonstrated that both older adults and younger adults can manage their increased anxiety by employing a stiffness posture strategy. These findings are corroborated by the study by Hauck et al. [47], which offers fresh proof that shifting task difficulty can similarly affect balance efficacy, anxiety, and stability. In this context, the observed slowing suggests that the stressor negatively affected the speed and coordination of dynamic movement [18].

On the other hand, as Thomas & Lane [48] point out, the TUG has predictive capacity to detect future falls because components such as sitting and standing, walking, and turning require many aspects of postural control. Likewise, the TUG can differentiate between people who have suffered falls and those who have not [49]. However, the predictive validity of the TUG test score for identifying future falls must be contextualized according to the sample [27].

In this study, the baseline mean of 7.00 ± 1.16 s and the post-mean of 7.37 ± 1.40 s indicate a sample of highly functional adults. Compared to other studies that have also assessed the TUG under safe and rapid execution orders, the participants in the present study performed above average for their age range (which, according to the systematic review and meta-analysis by Long et al. [50], is 8.67 s for people aged 70 to 79) and far from the usual fall risk thresholds of 11 to 13.5 s [25,27]. In the systematic review of Beauchet et al. [51], which analyzed the TUG independently of the pace at which the test was performed, the cut-off times ranged from 10 to 32.6 s. However, although the observed increase in time in our study was 0.37 s, in highly functional older adults, the mean difference in TUG time between fallers and non-fallers is only 0.63 s [27]. Therefore, this 0.37 s delay uses up nearly 60% of the small margin (0.63 s) that separates fallers from non-fallers in high-functioning samples. These results are consistent with the study by Möller et al. [17], which demonstrated that emotional stress acts as an acute trigger, thereby increasing the risk of falls for up to one hour after the stressor. Thus, the results of the present study suggest that emotionally stressful stimuli could compromise dynamic functional mobility and increase the immediate risk of falls, even in highly functional older adults. Future studies should evaluate the impact of these emotionally stressful stimuli on TUG performance in older adults of different age ranges and with distinct levels of functionality.

The results could potentially be explained by variations in the TUG’s administration and testing settings. In the original publication’s methodology section, Podsiadlo & Richardson described walking at a comfortable and secure speed [21]. However, like other studies, ours used fast-paced walking [25,52,53]. Although using a fast pace deviates from the test’s initial description, compared to studies that employed a usual-pace format, the test’s performance under this circumstance does not alter the nature of the link with falls [51].

The sample in this study consisted of a greater number of women than men. Women may report higher levels of chronic baseline stress [54]. However, previous studies examining the immediate response to a stressor have shown that the physiological response mechanism and subjective assessment of stress are comparable between the two sexes in older adults [55]. Since the objective of the present study was to explore differences in the TUG in response to an acute stressful emotional stimulus, a joint analysis of the sample was performed. On the other hand, the sample size (n = 31) was adequate to characterize the differences observed in the TUG test performance in response to the emotional stimulus. The a priori power calculation ensured 85% sensitivity to detect an effect size of d = 0.60. The observed effect was greater (d = 0.76), and the results were highly significant (*p* < 0.001). Notably, this design exceeded the statistical power of the reference study by Akinlosotu et al. [18], which analyzed 22 participants to achieve a standard power of 80% to detect changes in balance under stressful conditions in older adults.

This study presents several limitations that must be considered. Firstly, the cross-sectional design does not allow for establishing causal relationships and lacks a control group, limiting the ability to distinguish whether TUG changes are specifically due to stress or to factors such as fatigue or learning effects. However, for learning to occur, a greater number of repetitions than those performed in this study [56] would be necessary, and learning would have resulted in a reduction in TUG time rather than an increase as observed. Likewise, the rest period between the first TUG performance and exposure to the stimulus should have prevented fatigue. Secondly, participants present baseline TUG times indicating low fall risk from the outset, which limits the generalizability to older adults with higher fall risk or a history of falls. Thirdly, although all participants were non-institutionalized individuals living in the community, the study has the limitation of not having explored the specific family context (e.g., living alone or with others) or detailed socioeconomic status. These variables can influence coping capacity and the impact of stressful stimuli [54] and should be analyzed in future studies. Lastly, the use of the modified fast-paced TUG reduces comparability with standard protocols and published normative values and can limit clinical decisions. The observed change (0.37 s) is statistically significant but could be clinically modest in a population with very low fall risk. However, this research highlights the importance of assessing the autonomic nervous system performance under stressful conditions in elderly people to deepen the understanding of dynamic functional mobility.

## 5. Conclusions

The study demonstrated that acute emotional stimulation increased subjective indicators of discomfort and decreased parasympathetic dominance, confirming the successful induction of stress.

Performance on the TUG test at fast and safe speeds deteriorated statistically significantly after the stimulus, indicating a reduction in dynamic functional mobility. This suggests that exposure to an emotionally stressful stimulus could compromise dynamic functional mobility and increase acute fall risk in older adults.

## Figures and Tables

**Table 1:** Analysis of secondary outcome variables.

Variable	Statistical Test	Pre-Stress (M ± SD/ Md [Q_1_; Q_3_])	Stress (M ±SD/ Md [Q_1_; Q_3_])	Statistic	95% CI	*p*-Value	Effect Size
HF (ln)	*t*-test	3.54 ± 0.5	3.19 ± 0.61	t(30) = 3.64	[0.15, 0.55]	0.001	d = 0.653
LF	Wilcoxon	65 [48.6; 77.5]	77.7 [64.5; 85.1]	Z = −3.11	[−14.6, −2.69]	0.002	r = 0.559
LF/HF	Wilcoxon	1.89 [0.95; 3.49]	3.49 [1.82; 7.74]	Z = −2.87	[−2.45, −0.3]	0.004	r = 0.515
VAS	Wilcoxon	1.1 [0; 1.75]	6 [2.65; 8.5]	Z = −4.54	[−5.65, −2.85]	<0.001	r = 0.816

CI: confidence interval; HF: high-frequency; LF: low-frequency; ln: logarithmic transformation; M: Mean; Md: Median; SD: standard deviation; VAS: visual analogue scale.

## Data Availability

The dataset is available upon request from the authors.

## Abbreviations

The following abbreviations are used in this manuscript:

DEPIE	Use of Devices for Detecting Balance in Older Adults under Stress Influence
HF	High-frequency
HRV	Heart Rate Variability
IAPS	International Affective Picture System
LF	Low-frequency
M	Mean
Md	Median
SD	Standard Deviation
TUG	Timed Up and Go
VAS	Visual Analogue Scale
WHO	World Health Organization
